# Acid-Resistance Enhancement of Thin-Film Composite Membrane Using Barrier Effect of Graphene Oxide Nanosheets

**DOI:** 10.3390/ma14123151

**Published:** 2021-06-08

**Authors:** Hee-Ro Chae, In-Chul Kim, Young-Nam Kwon

**Affiliations:** 1 Center for Membrane, Advanced Green Chemical Materials Division, Korea Research Institute of Chemical Technology, Daejeon 34114, Korea; chr0619@snu.ac.kr; 2 School of Urban and Environmental Engineering, Ulsan National Institute of Science and Technology (UNIST), Ulsan 44919, Korea

**Keywords:** graphene oxide nanosheets, polyamide membrane, acid resistance, barrier effect

## Abstract

In this study, the effect of graphene oxide nanosheets (GONs) embedded in a thin-film composite (TFC) polyamide (PA) membrane on the acid resistance of the membrane was investigated by comparison with the effect of oxidized single-walled carbon nanotubes (o-SWNTs). Both GONs and o-SWNTs increased the hydrophilicity of the membranes and caused the formation of ridges and clustered bumps on the surfaces, resulting in slightly improved water permeability. However, the o-SWNTs-embedded membrane did not show a difference in acid resistance depending on the concentration of embedded material, but the acid resistance of the GONs-embedded membrane increased with increasing concentration. The acid resistance of the GONs-embedded membranes appears to be mainly due to the barrier effect caused by the nanosheet shape of the GONs along with a sacrificial role of the PA layer protruded by the addition of GONs and the decrease of acid reaction sites by the hydrogen bonding between GONs and PA. When the TFC PA membrane was prepared with a high amount (300 ppm) of the GONs without considering aggregation of GONs, membrane selectivity exceeding 95% was maintained 4.7 times longer than the control TFC membrane. This study shows that the acid resistance can be enhanced by the use of GONs, which give a barrier effect to the membrane.

## 1. Introduction

A nanofiltration (NF) membrane is a physical barrier that allows solvent and monovalent ions to permeate through but rejects divalent and multivalent ions. Due to the capability of the selective transport, NF has been widely utilized for various water/wastewater treatment and water softening. Recently, the demand for NF membranes in the field of acid processing of the metal [1], pulp [2], dairy [3], and mining [3] industries has been increasing, since NF can extract valuable and/or rare metals from mine and electroplating wastewater by retaining them in the feed stream and can purify the acid for reuse by only passing acid through the membrane, recovering useful resources, and satisfying environmental regulations [4,5].

However, membrane manufacturers suggest that the polyamide (PA) membrane should be used at pH 2 or higher [6], which means the durability of the membrane is deteriorated below this pH value. In particular, piperazine (PIP)-based PA membranes have been reported to be much less stable in acid than m-phenylenediamine (MPD)-based membranes. Jun et al. [7] conducted density functional theory (DFT) calculations and also investigated the physico-chemical property changes by the exposure of the PIP-based and MPD-based PA membranes to strong acid. They showed that protonation of oxygen in the PIP-associated moiety of the PA membrane is easiest among the protonation sites of the PIP-based and MPD-based membranes, and N-protonation of the PIP moiety has the lowest energy barrier in the rate determining step (RDS) of hydrolysis. In another study they showed that partial hydrolysis of the PA membrane by acid can be utilized to use the PA membranes in water softening and concentration of valuable antibiotics [8].

Many studies have been conducted to increase the acid resistance of PA thin-film composite (TFC) membranes. In the early research of related fields, studies were conducted mainly to evaluate the stability of commercial membranes in the presence of acids [5,9,10,11]. In recent years, research has been carried out to improve the acid resistance of TFC membranes, and related methods have been reported. Lee et al. [12] fabricated an acid-stable TFC membrane with a polyamine active layer. The polyamine contains no carbonyl groups, which are common reaction sites for initiation of hydrolysis, and hydrolysis of the active layer in an acidic condition was thereby suppressed. Liu et al. [13] increased the acid resistance of a commercial TFC membrane by coating the membrane surface with poly(N-isopropylacrylamide-co-acrylamide). The coating was achieved via circulation of an aqueous solution of the polymer in a crossflow system where the TFC membrane was installed.

Recently, studies using carbon-based materials to improve the permeability of PA TFC membranes have been reported. Jin et al. prepared a high performance polyamide membrane using GO aggregates, crosslinked and functionalized with m-xylenediamine, and trimethyl chloride (TMC) [14]. Sianipar et al. reported that, through an intensive review of researches related to carbon nanotubes (CNTs), functionalized CNTs used for membrane formation can avoid the trade-off issue between water flux and salt rejection [15]. Perreault et al. fabricated TFC PA membrane functionalized with graphene oxide nanosheets, imparting biocidal activity without negative effect on membrane transport properties [16]. A review of graphene material fabrication methods has been well presented recently [17] and studies on the structural factors of carbon composites on performance have been performed [18], making it easier to use carbon-based materials for membrane fabrication. In this study, graphene oxide nanosheets (GONs) were embedded in the PA layer of a TFC membrane to enhance the acid resistance of the PIP-based NF TFC membrane. The hypothesis of enhanced acid stability by addition of GONs was based on the results of previous research showing that chlorine resistance increased in a GONs-embedded PA membrane due to the barrier effect of the GONs and hydrogen bonding between GONs and PA [19]. In addition, another study reported that the acid resistance of the TFC membrane can be enhanced via those effects by coating copolymer on the PA layer [13]. Oxidized single-walled carbon nanotubes (o-SWNTs), which have a similar chemical composition to that of GONs, were also embedded in the PA layer of a TFC membrane to confirm the barrier effect of GONs indirectly. Salt rejection of those membranes immersed in 50% sulfuric acid was monitored and evaluated over exposure time.

## 2. Materials and Methods

### 2.1. Materials

Graphite (Sigma-Aldrich, St. Louis, MO, USA) and SWNTs (US Research Nanomaterials, Houston, TX, USA) were used as precursors for GONs and o-SWNTs, respectively. Sulfuric acid (H_2_SO_4_, Samchun Chemical, Seoul, Korea), nitric acid (HNO_3_, Samchun Chemical, Seoul, Korea), and potassium permanganate (KMnO_4_, Sigma-Aldrich, St. Louis, MO, USA) were employed to oxidize the precursors. Hydrogen peroxide (H_2_O_2_, Junsei Chemical, Tokyo, Japan) was used to reduce excess potassium permanganate. Sulfuric acid was also used for acid resistance tests. Piperazine (PIP, Samchun Chemical, Seoul, Korea), trimesoyl chloride (TMC, Sigma-Aldrich, St. Louis, MO, USA), and isoparaffin (ISOL-C, SK Chemical, Seoul, Korea) were used to fabricate a PA active layer of TFC membranes. In addition, a polysulfone (PSf) ultrafiltration membrane (MWCO 100,000, LG Chem., Seoul, Korea) was used as a support layer of the TFC membranes. Magnesium sulfate (MgSO_4_, Samchun Chemical, Seoul, Korea) was employed to evaluate membrane performance.

### 2.2. Preparation of GONs and o-SWNTs

GONs and o-SWNTs were prepared by oxidation of graphite and SWNTs based on the Hummers method [20]. The precursors were oxidized by oxidants such as sulfuric acid, nitric acid, and potassium permanganate. Residual excess potassium permanganate was then removed by hydrogen peroxide. The solutions containing the oxidized precursors were neutralized by replacing the acidic supernatant, obtained via centrifugation, with DI water several times. The graphitic oxides were then exfoliated by strong sonication using a sonication probe (VCX 750, Sonic Materials, Newtown, CT, USA) at 563 W for 2 h, and the o-SWNTs were dispersed by mild sonication using a sonication bath (JAC- 2010, Jinwoo-Alex, Seoul, Korea) at 200 W for 1 h.

### 2.3. Preparation of GONs- and o-SWNT-TFC Membranes

A PSf support layer was immersed in a 4 wt% PIP aqueous solution including GONs or o-SWNTs at a concentration of 0, 20, or 40 ppm for 1 min. The reason for using low concentration was to evaluate the effect of the embedding additives on acid resistance due to the shape of additives, excluding their aggregation effect, because the degree of aggregation is different at high concentration. Excess PIP solution remaining on the support layer was removed by squeezing it using a rubber roller for approximately 30 s under an air blowing condition. After drying in air for 30 s, the PSf support layer was then immersed in a solution of 0.2 wt% TMC in ISOL-C for 1 min to form a PA active layer via interfacial polymerization. Finally, the TFC membrane was annealed in an oven at 60 °C for 10 min and then stabilized in DI water for 90 min. The TFC membranes embedded with GONs or o-SWNTs are denoted as GONs- or o-SWNT-TFC membranes, respectively.

### 2.4. Characterization of GONs and o-SWNTs

To confirm the chemical compositions of GONs and o-SWNTs, C1s X-ray photoelectron spectroscopy (XPS) spectra were collected using an X-ray photoelectron spectrometer (AXIS NOVA, Kratos Analytical, Manchester, UK). The samples were prepared by dropping a dispersion of GONs or o-SWNTs onto a silicon wafer (4 WAFER P-100, Sehyoung Wafertech, Seoul, Korea) and then drying repeatedly.

### 2.5. Characterization of GONs- and o-SWNT-TFC Membranes

The presence of GONs or o-SWNTs in the PA layers of the prepared membranes was examined using a nano-Raman spectrometer (InVia, Renishaw, Wotton-under-Edge, UK). The Raman spectra of the TFC, GONs-TFC, and o-SWNT-TFC membranes were acquired in the range of 1000–2000 cm^−1^ using a 514 nm continuous wave laser. The contact angle of the TFC, GONs-TFC, and o-SWNT-TFC membranes was evaluated using a drop-shape analysis system (DSA 100, Krüss, Hamburg, Germany) through the sessile drop method with a 5.0 μL DI water drop after drying the membranes for 24 h. The surface morphology of the TFC, GONs-TFC, and o-SWNTs-TFC membranes before and after acid treatment was observed by field-emission scanning electron microscopy (FE-SEM, SIGMA HD, Carl Zeiss, Germany; JSM-6700F, JEOL, Tokyo, Japan) after coating platinum on the surface of the membranes with a sputter coater (Sputter Coater 108, Cressington, Watford, UK).

### 2.6. Evaluation on the Performance of GONs- and o-SWNT-TFC Membranes

The acid resistance of the TFC, GONs-TFC, and o-SWNTs-TFC membranes was evaluated by observing the changes in performance of the membranes after acid treatment. The salt rejection and the water flux of the membranes soaked in 50% sulfuric acid for a certain duration were measured using a 2000-ppm MgSO_4_ solution at 225 psi for 10 min after 30 min conditioning in a cross-flow filtration system. The effective membrane area and crossflow velocity were 27.9 cm^2^ and 19.6 m/min, respectively. The temperature of the feed solution was maintained at 25 °C using a refrigerated circulating water bath (RW-0525G, Lab Companion, Daejeon, Korea).

## 3. Results

### 3.1. Characterization of GONs and o-SWNTs

**Figure 1:**
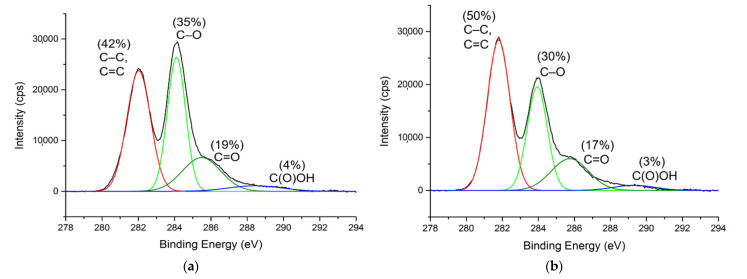
C1s XPS spectra of (**a**) GONs and (**b**) o-SWNTs.

The C1s XPS spectra of GONs and o-SWNTs (Figure 1) were deconvoluted into four gaussian sub-peaks: C–C and C=C (~281.9 eV), C–O (~284.0 eV), C=O (~285.6 eV), and C(O)OH (~288.9 eV) [21,22]. The spectra of GONs and o-SWNTs appear similar, but the ratio of peak height 281.9 eV to 284.0 eV was different between the two cases. The C1s XPS spectrum of o-SWNTs (Figure 1b) has a larger peak at ~281.9 eV and a smaller peak at ~284.0 eV, indicating that the o-SWNTs contained more C–C and C=C functional groups and fewer C–O functional group than the GONs. That is, the o-SWNTs (50% oxidized) were less oxidized than the GONs (58% oxidized).

### 3.2. Characterization of GONs- and o-SWNTs-TFC Membranes

**Figure 2:**
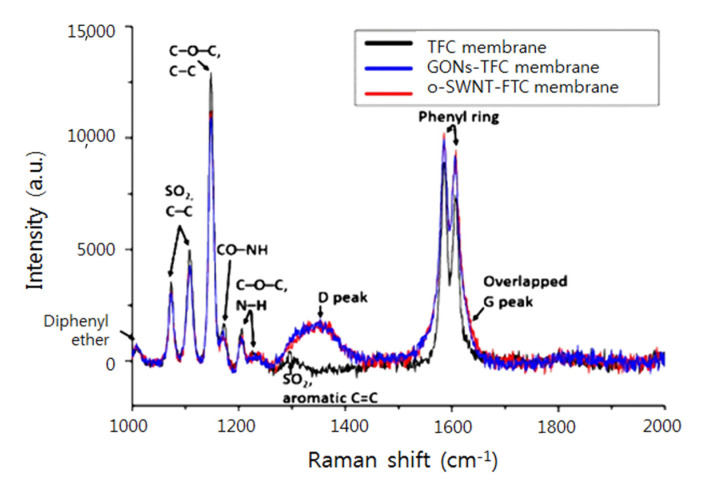
Raman spectra of TFC (black line), GONs-TFC (blue line), and o-SWNTs-TFC (red line) membranes.

The presence of GONs and o-SWNTs in the PA layer of the membranes was investigated using Raman spectroscopy (Figure 2). The Raman spectrum of the TFC membrane (black line) exhibits typical peaks of PSf and PA [23,24,25,26,27]. The very weak peak at 1007 cm^−1^ originates from symmetric stretching of diphenyl ether. The two peaks at 1073 and 1108 cm^−1^ correspond to symmetric and antisymmetric SO_2_ stretching, respectively, and also C–C stretching. The one strong peak at 1148 cm^−1^ originates from symmetric C–O–C and C–C stretching, and the overlapping weak peak at 1171 cm^−1^ corresponds to CO–NH skeletal motion. The weak peak at 1206 cm^−1^ and the weak broad peak at 1226 cm^−1^ arose from asymmetric and antisymmetric C–O–C stretching and N–H wagging, respectively, and the very weak broad peak at 1295 cm^−1^ came from asymmetric SO_2_ stretching and symmetric aromatic C=C stretching. The two strong peaks at 1585 and 1606 cm^−1^ correspond to phenyl ring vibrations.

The Raman spectra of the GONs- (blue line) and o-SWNT-TFC (red line) membranes are similar to the spectrum of the TFC membrane, with the exception of the broad peak at 1350 cm^−1^ and the double peaks at ~1600 cm^−1^, which could indicate the addition of a broad peak. Two such broad peaks are typical of GONs and o-SWNTs, and are called D and G peaks, respectively [28,29]. The D and G peaks are from the in-plane stretch vibrations of aromatic rings, and in particular, the D peak only appears in the presence of (graphite lattice) disorder [28]. The observation of D and G peaks in Figure 2 confirms the presence of GONs and o-SWNTs in the GONs- and o-SWNT-TFC membranes, respectively.

**Figure 3:**
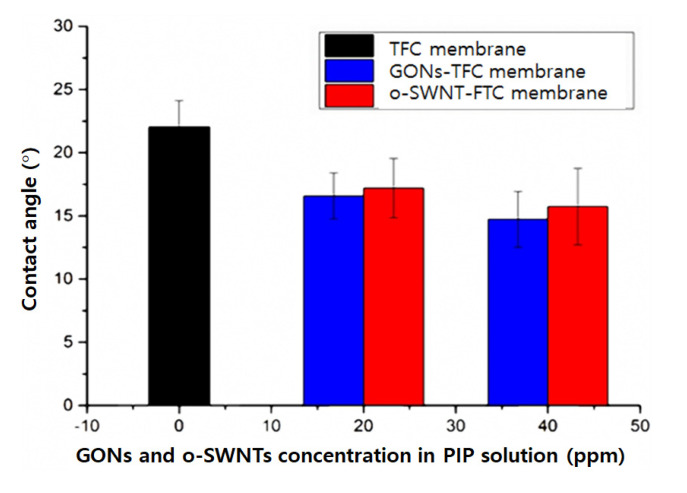
Contact angle of TFC, GONs-TFC, and o-SWNTs-TFC membranes (*n* = 8).

The contact angle of the TFC membrane decreased slightly after embedment of GONs or o-SWNTs (Figure 3) into the PA layer. The contact angle of the GONs-TFC membrane was only marginally smaller than that of the o-SWNT-TFC membrane within the margin of error. In other words, the hydrophilicities of the GONs- and o-SWNT-TFC membranes do not noticeably differ but are higher than that of the TFC membrane.

**Figure 4:**
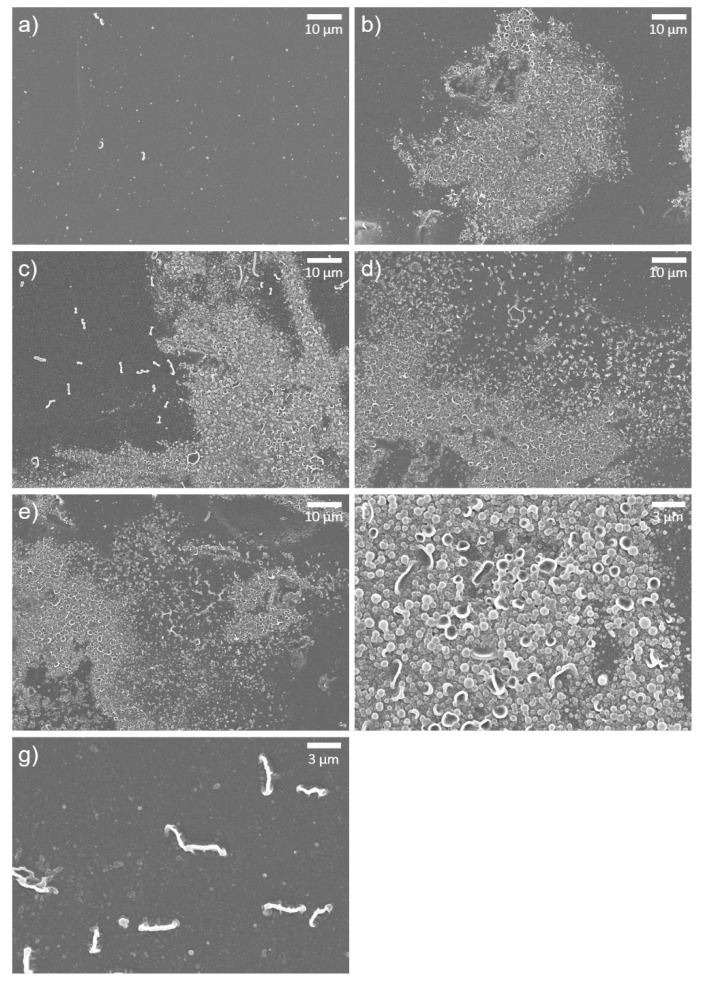
SEM images of (**a**) TFC, (**b**) GONs-TFC (20 ppm), (**c**) GONs-TFC (40 ppm), (**d**) o-SWNTs-TFC (20 ppm), and (**e**) o-SWNTs-TFC (40 ppm) membranes at 3000× magnification, (**f**) bumps and (**g**) ridges of a GONs-TFC membrane (40 ppm) at 10,000× magnification. These figures show the surface of the membranes before exposure to acid solution.

The surface morphology of the TFC, GONs-TFC, and o-SWNTs-TFC membranes was observed by SEM. The surface of the TFC membrane (Figure 4a) was very smooth and had a few tiny ridges, whereas the surfaces of the GONs- and o-SWNTs-TFC membranes (Figure 4b–e) had clusters of bumps (white protruding region in Figure 4b–f and many tiny ridges (thick white protruded lines in Figure 4b–e,g). The clusters of bumps were formed on almost half of the surface. The bumps (Figure 4f) had convex or concave shapes. The head of a tiny concave bump was similar to the convex bump. Therefore, the convex bump might be an early form of the concave bump. These morphologies could be caused by differences in the solubility of PIP, GONs, and o-SWNTs in the organic solvent. As is well known, diffusion of amine monomers (e.g., PIP) into an organic solvent, which causes the formation of bumps and ridges [30], is related to solubility in the solvent [31]. A monomer that is more soluble in the organic solvent can form larger and more bumps and ridges on a membrane surface. Meanwhile, as the solubility of GONs in the organic solvent is much greater than that of PIP [32,33], PIP might be dragged by the GONs toward the organic solvent owing to faster diffusion of the GONs. Therefore, the formation of ridges and bumps on the PA layer could be promoted. o-SWNTs, which have a similar chemical composition to that of GONs, should also have analogous solubility in an organic solvent. Thus, the addition of o-SWNTs to a solution of PIP could also cause protuberance on the PA layer. The bumps in the figures were not attributed to the aggregation of GOs, since wall-like wrinkles are formed on the membrane surface when GOs are aggregated.

**Figure 5:**
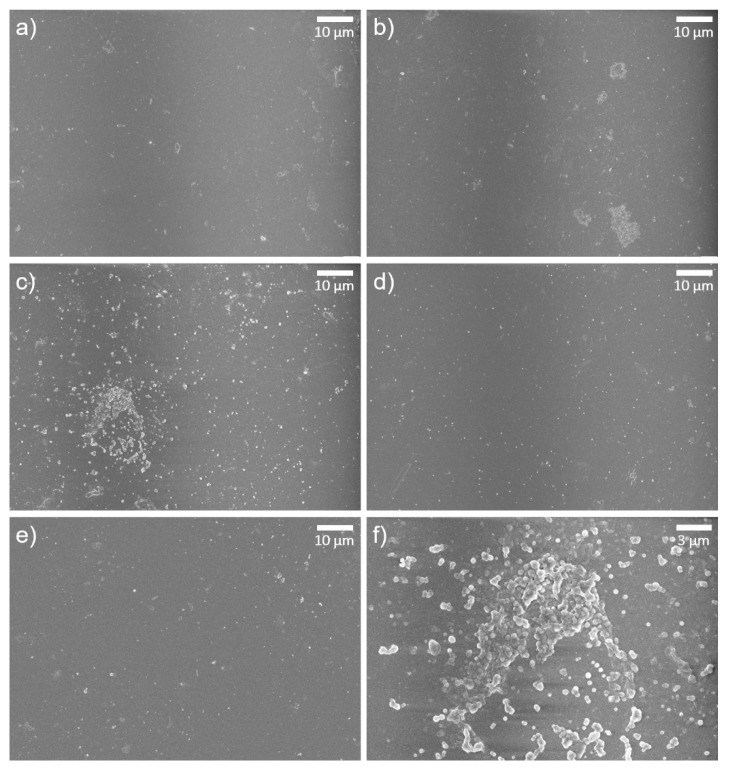
SEM images of (**a**) TFC, (**b**) GONs-TFC (20 ppm), (**c**) GONs-TFC (40 ppm), (**d**) o-SWNTs-TFC (20 ppm), and (**e**) o-SWNTs-TFC (40 ppm) membranes at 3000× magnification, and (**f**) a GONs-TFC membrane (40 ppm) at 10,000× magnification. These images were acquired after the acid resistance test.

The surface morphologies of the membranes after exposure to acid were also investigated (Figure 5). The control TFC membrane (Figure 5a) had a very smooth surface, similar to that before the exposure to acid, indicating that there is no morphological difference before and after acid treatment. The surface of the o-SWNTs-TFC membrane was also smoother after acid treatment, but tiny white spots existed on the surface (Figure 5d,e). They might be vestiges of ridges or bumps. This observation suggested that the top of the o-SWNTs-TFC embedded PA layer including bumps and ridges was degraded considerably. However, on the GONs-TFC membrane, a few small clusters of bumps (white protruded regions in Figure 5b,c,f) withstood the acid treatment. In detail, the shape of the clusters of bumps (Figure 5f) was different from that before acid treatment (Figure 4f). Each bump seems to melt down, and the bumps were connected to each other. That would be the result of degradation by sulfuric acid. The PA active layer of the GONs-TFC membrane was likely maintained.

**Figure 6:**
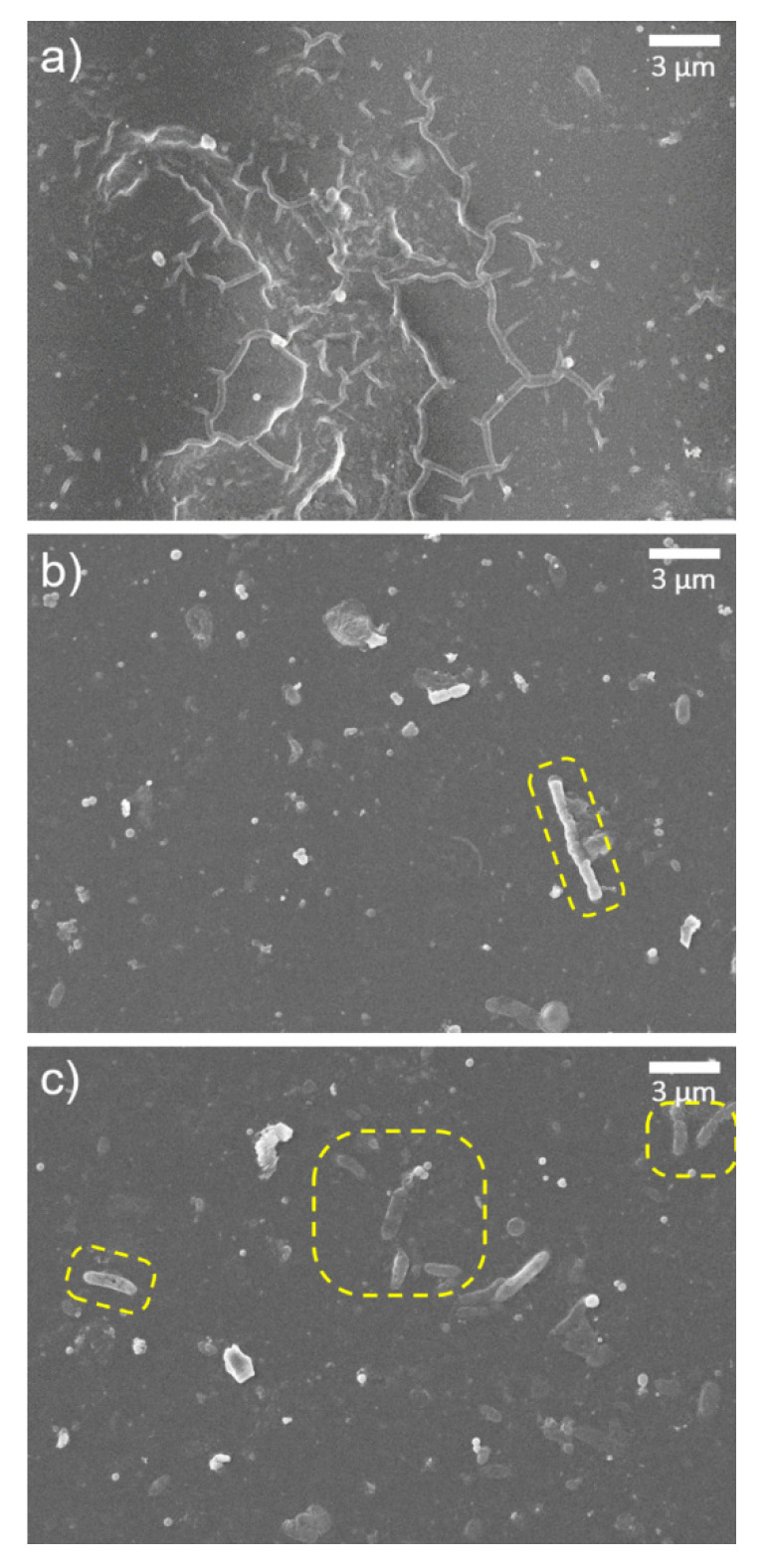
SEM images of (**a**) a wrinkled pattern on a GONs-TFC membrane (40 ppm) and (**b**) something that seems to be o-SWNT and (**c**) microbes on an o-SWNT-TFC membrane (40 ppm). These images were acquired at 10,000× magnification after the acid resistance test.

After acid treatment, several things that look like GONs and o-SWNTs, or microbes were found on the membrane surface (Figure 6). In detail, the wrinkled pattern was observed on the GONs-TFC membrane because the upper part of the PA layer was degraded (Figure 6a). Such patterns are typical of GONs, whether they are embedded in a PA layer [19] or not [34]. The yellow dashed regions in Figure 6b seem to be rod-shaped o-SWNTs on the surface of the o-SWNT-TFC membrane. The width of the wrinkle and rod are several dozen times thicker than GONs and o-SWNTs, respectively. That could be due to the wrapping of the o-SWNTs by PA. The structure looked like o-SWNTs are thinner, brighter, and less rounded [35], but the microbes are relatively thicker, darker, and more rounded [36].

### 3.3. Evaluation of the Performance of GONs- and o-SWNTs-TFC Membranes

**Figure 7:**
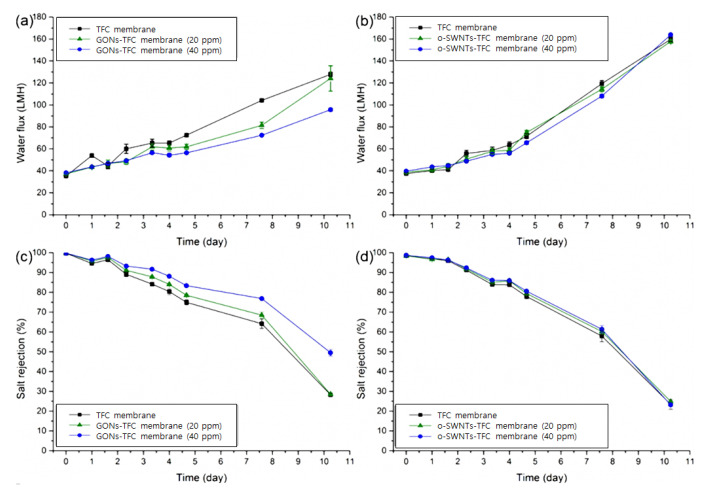
Water flux of (**a**) GONs- and (**b**) o-SWNTs-TFC membranes (0, 20, 40 ppm) and salt rejection of (**c**) GONs- and (**d**) o-SWNTs-TFC membranes (0, 20, 40 ppm) with a 2000-ppm MgSO_4_ feed solution. The time in the *x*-axis represented the time of immersion in sulfuric acid (*n* = 3).

Figure 7 shows the changes in water flux and salt rejection of the TFC, GONs-TFC, and o-SWNTs-TFC membranes exposed to acid under the condition of a 2000-ppm MgSO_4_ feed solution. Before acid exposure, water flux of the membrane was 8% (for 40 ppm GONs) or 6% (for 40 ppm o-SWNTs) higher than that of the control membrane owing to enhanced hydrophilicity and roughness caused by local protrusions [37,38]. The effect of GONs and o-SWNTs on the water flux of the PIP-based NF membrane was smaller than that observed in previous studies using MPD, which is likely because the hydrophilicity of PIP-based PA is much higher [39,40] than that of MPD-based PA [41,42].

Over time, salt rejection of the TFC membrane decreased with acid treatment, but the decline rate was reduced by the embedment of GONs. At the end of the test, the GONs-TFC membrane (40 ppm) exhibited 21% higher salt rejection than the TFC membrane. This acid resistance might originate from the (physical) barrier effect of GONs and hydrogen bonding between GONs and PA. The barrier effects have been previously reported for chlorine resistance of TFC membranes [19,43,44] and for enhanced acid resistance of TFC membranes coated with another material [13]. In detail, GONs would lie horizontally on the PA layer such as in the case of Langmuir–Blodgett film deposition [45,46], indicating horizontal deposition of films on a substrate when a substrate is pulled out vertically from a solution containing film. In this study, the support layer was pulled out vertically from a PIP solution including GONs. The horizontally arrayed GO nanosheets could act as a physical barrier, protecting the underlying PA against acid solution [19,43]. Moreover, the functional groups of GONs could form hydrogen bonds with the carbonyl groups of PA [47]. Such hydrogen bonding could impede the addition of hydrogen to carbonyl groups, the initiating site of hydrolysis [13]. In addition, the bumps on GONs- and o-SWNT-TFC membranes (Figure 4b–e) might act as a sacrificial layer to further increase their acid resistance [48].

However, the embedment of o-SWNTs did not effectively suppress the decrease in salt rejection of the membrane by acid solution exposure, unlike the case of GONs, although the performance and properties of the GONs- and o-SWNT-TFC membranes were almost the same as before the acid treatment. This difference in the behavior of the GONs- and o-SWNT-TFC membranes could be attributed to the different shapes of GONs and o-SWNTs. Though very similar in composition [49], GONs and o-SWNTs, respectively, have planar and rod-like shapes. The projection area of o-SWNT is ~32% (calculated) smaller than that of GONs, and the curved surface and narrow width (OD: ~1.5 nm) of the o-SWNT could allow to make a detour to the underlying PA. Therefore, the acid resistance of the o-SWNTs-TFC membrane only marginally increased because o-SWNTs could only make a sacrificial layer and hydrogen bonds with PA without a sufficient barrier effect. Consequently, the barrier effect is likely the primary cause of the acid resistance of the GONs-TFC membrane compared with the o-SWNTs-TFC membrane.

**Figure 8:**
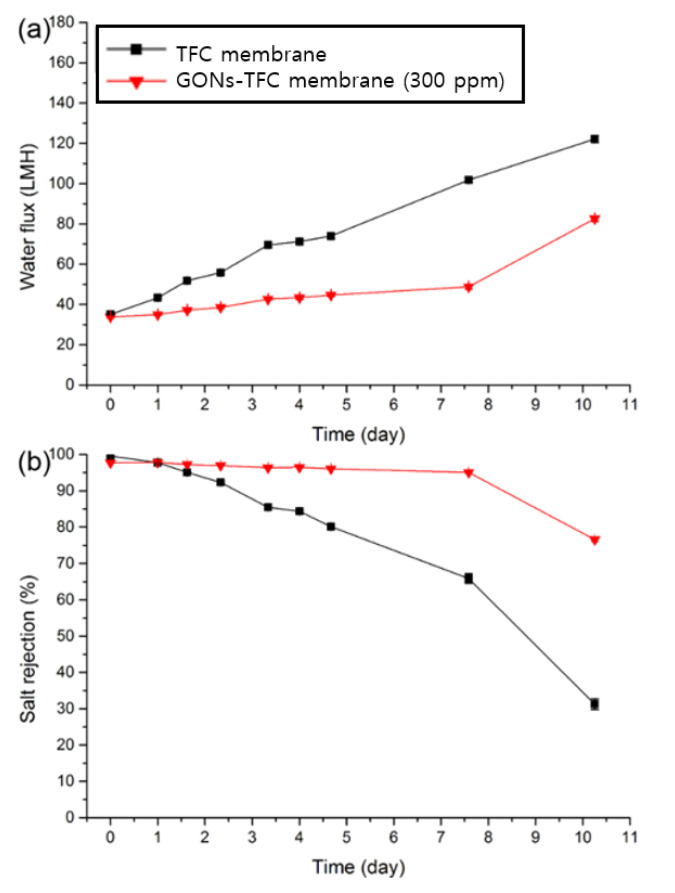
(**a**) Water flux and (**b**) salt rejection of TFC and GONs-TFC (300 ppm) membranes with a 2000-ppm MgSO_4_ feed solution. The time in the *x*-axis represented the time to immerse in sulfuric acid (*n* = 3).

Furthermore, the concentration of GONs in the PIP aqueous solution was increased to 300 ppm to evaluate the acid resistance of the GONs-TFC membrane without excluding severe particle aggregation during the preparation of the NF membrane. The GONs-TFC membrane did not contain any other additives to enhance acid resistance without GONs for clear analysis. The preparation and test conditions were the same as the above conditions (50% sulfuric acid). The decline in salt rejection was retarded more using the 300 ppm GONs solution (Figure 8b) than using the 40 ppm GONs solution (Figure 7c). The GONs-TFC membrane (300 ppm) maintained good salt rejection (>95%) 4.7 times longer than the control TFC membrane.

## 4. Conclusions

A small amount of GONs embedded in the PA layer of a TFC membrane improved the acid resistance of the TFC membrane, and the improvement is believed to be caused by the barrier effect of GONs, hydrogen bonding between GONs and PA, and the formation of a sacrificial role of PA layer protruded by GONs. However, o-SWNTs, which have a similar chemical composition to GONs, exhibited only a small effect on the acid resistance of the TFC membrane because o-SWNTs could not provide a barrier effect owing to their rod-like shape. Therefore, the barrier effect is likely the primary cause of the acid resistance of the GONs-TFC membrane. This study shows that the acid resistance can be improved by the use of additives that provide a barrier effect such as GONs. The improvement in the acid resistance is expected to broaden the use of polyamide membranes in separation, purification, and concentration processes for various solutions including strong acids.

## Data Availability

The data underlying this article will be shared on reasonable request from the corresponding author.
